# Essential worker status, gender, and migration background disparities in COVID-19: An intersectional approach

**DOI:** 10.1016/j.jmh.2025.100392

**Published:** 2025-12-27

**Authors:** Narges GHOROUBI, Myriam KHLAT, Emilie COUNIL, the EpiCoV Study group

**Affiliations:** a The French Institute for Demographic Studies (Ined), 9 Cours des Humanités, 93322 Aubervilliers, France; b Doctoral School of Public Health (EDSP), Paris Saclay University, 16 avenue Paul Vaillant Couturier, 94807 Villejuif, France; c Institut de Recherche Interdisciplinaire sur les Enjeux Sociaux - Sciences sociales, politique, santé, IRIS (UMR 8156 CNRS - EHESS - U997 INSERM), 5 Cours des Humanités, 93322, Aubervilliers, France; d Nathalie Bajos, Josiane Warszawski, Guillaume Bagein, François Beck, Emilie Counil, Florence Jusot, Nathalie Lydie, Claude Martin, Laurence Meyer, Ariane Pailhé, Philippe Raynaud, Alexandra Rouquette, Delphine Rahib, Patrick Sicard, Rémy Slama, Alexis Spire, France

**Keywords:** SARS-CoV-2, Gender disparities, Immigrants’ health, Intersectionality, Essential workers, EpiCoV

## Abstract

**Objectives:**

COVID-19 disproportionately hit women, immigrants, ethno-racial minorities, and essential workers. This study examines disparities in SARS-CoV-2 seropositivity by gender and migration background in France (late 2020) and assesses how much essential worker status explains them.

**Methods:**

We grouped 30,018 workers from the EpiCoV cohort into four categories defined by gender and migration background. Worker status included seven categories: non-essential workers; high- and low-class healthcare workers (HCWs), social and educational workers (SEWs), and other essential workers (OEWs). We assessed SARS-CoV-2 seroprevalence across gender and migration-background groups. Non-linear decomposition analysis quantified the extent to which significant seropositivity differences were driven by unequal representation in essential occupations and varying infection risk within similar essential occupations.

**Results:**

SARS-CoV-2 seropositivity was lowest among men without a migration background, higher among women without a migration background, and highest among individuals with a migration background. Compared to men without a migration background, seropositivity was 1.6 percentage points higher among women without a migration background (43.7% attributed to overrepresentation among HCWs and 5.8% to greater risk within low-class HCWs), and 6.9 percentage points higher among women with a migration background (31.5% driven by their overrepresentation among HCWs and low-class SEWs, and 19.4% by stronger risks across low-class essential jobs). The 5.3-point seropositivity gap between women with and without a migration background was 33.4% attributable to stronger infection risks among low-class SEWs and low-class OEWs.

**Conclusions:**

Women bore a double burden exacerbated by their migration background: overrepresentation in certain essential jobs and elevated COVID-19 risk within these occupations.

## Highlights

• SARS-CoV-2 seropositivity was greater in women and those with migration background.
• Essential worker status explained part of women’s higher infection risk.
• Women were over-represented in health, social, and educational essential jobs.
• Women also had a higher within-job infection risk in some low-class essential jobs.
• This dual burden in women was intensified by migration background.

## 1 Introduction

The relationship between gender and COVID-19 infection risk in the general population remains inconclusive, with large, multi-country meta-analyses reporting both an increased risk in men (Abate et al., 2020; Pijls et al., 2021) and no difference between men and women (Bobrovitz et al., 2021). However, among working-age adults, women showed higher infection risk (Doerre and Doblhammer, 2022). Also, immigrants and racial minorities in Europe faced a higher risk of SARS-CoV-2 infection compared to native-born and white populations (Jaljaa et al., 2022). In France, this trend was notable among first-generation non-European immigrants, whereas the second generation did not show higher seropositivity rates (Warszawski et al., 2022b).

While non-occupational factors, such as household exposure, neighborhood conditions, and leisure activities, contribute to infection risk (McKee et al., 2024), occupational factors, especially essential worker status and occupational class, have likely played a central role in shaping such disparities. Indeed, essential workers faced higher exposure (Ghoroubi et al., 2022), which translated into significantly higher infection rates among healthcare workers early in the pandemic, when protective measures were still limited. For other essential occupations, most studies reported an increased risk, while others found no significant difference or even a lower risk (Rhodes et al., 2024). Importantly, essential workers within the same sector span a range of social classes with different risk levels; for example, healthcare cleaners were found to have nearly three times the infection risk of other healthcare workers (Bansal et al., 2025).

Two mechanisms may explain how holding an essential job may contribute to disparities by gender and migration background: compositional differences and association differences. Compositional differences refer to the unequal distribution of subgroups across occupational categories. There is evidence that women were disproportionately represented in the healthcare, education, and social services sectors, while men dominated other essential sectors such as transportation, construction, and repair (Blau et al., 2020). In France, women were overrepresented among essential workers, particularly in the healthcare sector (Ghoroubi et al., 2022). Across Europe, migrants were often overrepresented among essential workers (Fasani and Mazza, 2020). In France, the proportion of immigrant essential workers matches their share of total employment. However, certain essential occupations, such as cleaners, home helpers, and hospital doctors, rely heavily on immigrant labour (Edo et al., 2022). This overrepresentation also varied across occupational hierarchies. For example, in the U.S., women comprise 86 % of healthcare support jobs (e.g., nursing assistants, home health aides, hospital cleaners) and 75 % of practitioner and technical jobs (e.g., doctors, registered nurses, pharmacists), the latter typically requiring higher education and offering better wages (Blau et al., 2020). Similarly, immigrant workers in this country are overrepresented in healthcare support jobs while being proportionally represented in healthcare practitioner jobs. In particular, immigrant women are disproportionately found in healthcare support and cleaning jobs (Small et al., 2024). A comparable trend appears in France: women occupy 80 % of assistant-pharmacist posts but only half of titular roles, and 74 % of laboratory assistant director posts versus 40 % of director roles (Bessière, 2005).

The second mechanism, association differences, indicates that even within the same occupational category, some groups face a higher infection risk. Evidence supports the existence of gender-based task segmentation, whereby men and women perform different duties under the same job title, reflecting broader gendered labor patterns (Biswas et al., 2021). Working conditions can also differ; for instance, although women constitute most healthcare staff, personal protective equipment is typically modelled on male facial dimensions, making a proper fit, and therefore effective protection, harder for many women (Ascott et al., 2021).

A similar dynamic may apply to immigrant workers. Existing research from Europe and Canada suggests that they are more likely to have precarious contracts and may experience different risks despite holding the same job as natives due to differences in working conditions or exposures (Sterud et al., 2018). These factors could result in different workplace exposures and occupational risks, even within the same job title.

This literature overview highlights that prior research often examined these explanatory factors separately and under the assumption of "all else being equal." An intersectional approach (Bauer, 2014) can offer a more nuanced understanding of compounded vulnerabilities, such as those faced by immigrant women in low-class essential jobs, and thereby inform more effective and targeted public health interventions and policy design.

This study investigates differences in SARS-CoV-2 seropositivity by gender and migration background in metropolitan France at the end of 2020. It then assesses the extent to which these disparities can be explained by essential worker status, distinguishing between high- and low-class essential workers. Two mechanisms are explored: first, whether specific subgroups, such as women with a migration background, are more likely to occupy specific types of essential jobs; second, whether the association between different essential worker statuses and SARS-CoV-2 seropositivity varies across subgroups.

## 2 Methods

### 2.1 Database

We used the longitudinal, population-based EpiCoV cohort established in May 2020 in France. Participants aged 15 years and older were randomly selected from the national tax register, which covers 96 % of the population. Data were collected over four waves via computer-assisted web or telephone interviews, covering socio-demographic characteristics, socioeconomic status, health status, working conditions, living conditions, and COVID-19 outcomes. Details on sampling design and data collection are available elsewhere (Warszawski, Beaumont, et al., 2022).

### 2.2 Outcome variable: serology-confirmed SARS-CoV-2 infection

During the second wave of EpiCoV in autumn 2020, participants were invited to SARS-CoV-2 serology testing using a home blood sampling kit. Dried blood spot samples were analyzed for antibodies against the spike protein's S1 domain using a commercial enzyme-linked immunosorbent assay (ELISA) kit. We pooled the suspicious serologies (optical density ratio between 0.7 and 1.1) with the positive ones (optical density ratio greater than 1.1) due to the potential antibody decline over time (Gallais et al., 2021). Notably, 94.4 % of blood samples were collected before January 2021, preceding France's vaccination campaign, minimizing the risk of vaccine-induced seropositivity. Detailed methodology for the serology testing is available elsewhere (Warszawski, Beaumont, et al., 2022).

### 2.3 Study sample and weighting

The analysis included participants aged 18 to 64 living in metropolitan France who reported being employed (full-time or part-time) before the first lockdown and who participated in serological testing during the second wave of EpiCoV (*N* = 30,018; see Supplementary Fig. 1). Specific weights were calculated for participants who provided a serology sample, accounting for unequal sampling probabilities, differential non-response, and acceptance and return of the home-testing kit, and were calibrated to match known French population distributions from census and administrative data. The EpiCoV weighting protocol is detailed elsewhere (Bagein et al., 2022; Warszawski, Beaumont, et al., 2022). These weights were used throughout the analyses to ensure nationally representative estimates.

### 2.4 Explanatory and control variables

The main explanatory variable combined gender and migration background. In the absence of a dedicated gender question in EpiCoV, we had to rely on a binary sex variable, which we interpreted as gender. Participants were classified as having or not having a migration background. Individuals with a migration background included those born in French Overseas Departments (FOD; hereafter referred to as FOD natives), immigrants, and descendants of immigrants, based on participants’ and their parents’ place of birth and nationality at birth, following the definition used in the Trajectories and Origins survey (Beauchemin et al., 2019). This resulted in four groups: men without a migration background, women without a migration background, men with a migration background, and women with a migration background.

To investigate occupational explanations for COVID-19 disparities, we classified participants according to essential worker status and occupational class. Essential worker status included healthcare workers (HCWs), social and educational workers (SEWs), and other essential workers (OEWs), while all other occupations were classified as non-essential. Definitions for HCWs and SEWs were based on an official French classification (Acs et al., 2021), whereas OEWs followed the definition by Mutambudzi et al. (Mutambudzi et al., 2021) (see Supplementary Table 1). Occupational class was determined using the French classification system, which groups salaried and independent workers into four classes according to qualifications, job types, and contract conditions (Insee, 2022b). We combined the two highest classes into a "high-class" category and the two lowest classes into a "low-class" category. By combining essential worker status and occupational class, we created a seven-category variable.

The covariates included in the analysis were age (continuous), living in a densely populated neighborhood (binary), and living in overcrowded housing (binary) at inclusion. The variable "living in densely populated neighborhoods" separated densely populated municipalities from municipalities of intermediate, low, and very low density, as defined by Eurostat (Insee, 2022a), and overcrowded housing was defined as having a total number of bedrooms and living rooms lower than the number of household members.

### 2.5 Statistical analysis

We conducted three main statistical analyses:

First, we investigated compositional differences in the distribution of essential worker categories across gender and migration background subgroups. We fitted a multinomial logistic regression with the seven‐level occupational classification as the outcome (reference: non-essential workers) and the combined gender and migration background categories as the primary predictor (reference: men without a migration background). From the fitted model, we used the marginaleffects R package (Arel-Bundock et al., 2024) to compute average predicted probabilities for each occupational category within the gender and migration background subgroups as well as the overall sample, thereby estimating the expected proportion of individuals in each occupational category under the model.

Second, we examined association differences, defined as the variation in the strength of association between occupational categories and infection risk across gender and migration background groups. We stratified the data into four strata (men without a migration background, women without a migration background, men with a migration background, and women with a migration background) and ran separate logistic regression models for each, as well as one for the overall sample. The outcome variable was serology-confirmed SARS-CoV-2 infection, and the primary predictor was the essential worker status. This approach allowed us to calculate the relative risk of SARS-CoV-2 seropositivity for each essential worker category compared to non-essential workers within each stratum.

Third, we conducted a non-linear decomposition analysis using R functions developed by Daniel Powers (Powers, 2023/2024). This method extends the classical Oaxaca–Blinder framework to non-linear models, such as logistic regression, by decomposing group differences in terms of average predicted probabilities rather than raw means. It quantifies how much of the observed gap between two groups is due to differences in the distribution of risk factors and characteristics (compositional differences) versus differences in the strength of their association with health outcomes (association differences) (Powers et al., 2011).

In the decomposition models of this study, the outcome was SARS-CoV-2 seropositivity. The explanatory variable was the seven-category essential worker status and occupational class variable, and two covariates were included: living in a densely populated neighborhood and living in overcrowded housing. Group differences were defined by the combined gender and migration-background variable. We decomposed each statistically significant seropositivity gap observed between any two groups defined by gender and migration background. The analysis specifically separated two components: (a) how differences in the proportion of essential worker categories contribute to the seropositivity gap, assuming these categories have the same association with seropositivity as among the reference group (compositional differences); and (b) how differences in the association between essential worker status and seropositivity contribute to the gap, assuming the distribution of essential worker categories is the same as that of the reference group (association differences).

All descriptive analyses and models were conducted on complete cases, incorporating survey weights, and were conducted using R software.

### 2.6 Sensitivity analysis

We conducted two sensitivity analyses to assess the robustness of our findings.

First, we used a more specific definition of the outcome by restricting SARS-CoV-2 seropositivity to clearly positive ELISA results, defined as an optical density ratio greater than or equal to 1.1.

Second, given the absence of direct ethno-racial data in EpiCoV, we constructed an alternative binary migration-background variable to approximate visible minority status using the visible/invisible minority classification from the Trajectories and Origins survey (Beauchemin et al., 2019). This classification distinguished individuals without a migration background or invisible minorities from FOD natives or visible minorities. Due to the limited number of FOD native or visible minority men in certain occupational groups (only five participants in low-class SEW jobs), we combined low-class SEWs and OEWs into a single low-class non-healthcare essential worker category, and similarly merged high-class SEWs and OEWs into a high-class non-healthcare essential worker category to maintain analytical robustness.

## 3 Results

### 3.1 Sample description and seroprevalence of SARS-CoV-2

The weighted study sample had a nearly equal distribution of men and women, with 80 % of participants without a migration background and 20 % with a migration background, appropriately reflecting the working-age population living in France (DARES, 2023).

The majority of participants were non-essential workers, with relatively small proportions employed in essential occupations, including HCWs (5.0 % high-class, 4.4 % low-class), SEWs (6.1 % high-class, 4.9 % low-class), and OEWs (2.2 % high-class, 8.9 % low-class). Participants with a migration background, both men and women, were more likely to live in densely populated neighborhoods and overcrowded housing. Overall, 10.5 % of participants tested positive for SARS-CoV-2 antibodies. Seropositivity followed a gradient: 8.8 % in men without a migration background, 10.4 % in women without a migration background, 13.5 % in men with a migration background, and 15.7 % in women with a migration background. However, the gender difference among individuals with a migration background was not statistically significant (Table 1).

**Table 1:** Baseline characteristics of the study population in gender and migration background groups.

Variable			Men without a migration background *N* = 11,049	Women without a migration background *N* = 14,715	Men with a migration background *N* = 1910	Women with a migration background *N* = 2344	All *N* = 30,018	
			% [CI] / Mean (SD)	% [CI] / Mean (SD)	% [CI] / Mean (SD)	% [CI] / Mean (SD)	N	%[CI] / Mean (SD)
Share of participants			40.3 [39.5–41.2]	39.7 [38.9–40.5]	11.1 [10.3–11.8]	8.9 [8.4–9.4]		100
Essential worker and occupational class	Non-essential worker	77.6 [76.8–78.4]	57.6 [56.7–58.4]	79.7 [77.8–81.4]	62.8 [60.8–64.8]	20,576	68.5 [68–69.1]	
	HCW	High-class	2.5 [2.2–2.8]	8.4 [8–8.9]	1.7 [1.2–2.4]	5.5 [4.6–6.5]	1507	5.0 [4.8–5.3]
		Low-class	1.3 [1.1–1.6]	7.4 [7–7.9]	1.4 [0.9–2]	8.1 [7.0–9.3]	1309	4.4 [4.1–4.6]
	SEW	High-class	4.1 [3.7–4.4]	9.4 [8.9–9.9]	2.8 [2.1–3.7]	5.3 [4.4–6.2]	1845	6.1 [5.9–6.4]
		Low-class	0.6 [0.5–0.8]	9.1 [8.7–9.6]	0.8 [0.4–1.3]	10.5 [9.3–11.8]	1465	4.9 [4.6–5.1]
	OEW	High-class	3.4 [3–3.7]	1.4 [1.2–1.6]	1.6 [1.1–2.3]	1.1 [0.7–1.6]	653	2.2 [2.0–2.3]
		Low-class	10.6 [10–11.2]	6.7 [6.3–7.1]	12.1 [10.6–13.6]	6.8 [5.8–7.9]	2663	8.9 [8.6–9.2]
Living in a densely populated neighborhood	No		67.0 [66.2–67.9]	66.8 [66–67.6]	40.8 [38.6–43]	40.1 [38.1–42.1]	18,504	61.6 [61.1–62.2]
	Yes		33.0 [32.1–33.8]	33.2 [32.4–34]	59.2 [57–61.4]	59.9 [57.9–61.9]	11,514	38.4 [37.8–38.9]
Living in an overcrowded housing	No		93.7 [93.2–94.1]	93.9 [93.5–94.3]	75.6 [73.7–77.6]	83.5 [81.9–85]	27,284	90.9 [90.6–91.2]
	Yes		6.3 [5.9–6.8]	6.1 [5.7–6.5]	24.4 [22.4–26.3]	16.5 [15–18.1]	2734	9.1 [8.8–9.4]
Age	41.9 (11.3)		42.5 (11.2)	42.0 (11.6)	42.9 (11.0)		42.2 (11.2)	
Seropositivity	No		91.2 [90.7–91.8]	89.6 [89.1–90.1]	86.5 [84.9–88]	84.3 [82.8–85.8]	26,852	89.5 [89.1–89.8]
	Yes		8.8 [8.2–9.3]	10.4 [9.9–10.9]	13.5 [12–15.1]	15.7 [14.2–17.2]	3166	10.5 [10.2–10.9]

HCW: healthcare workers; SEW: social and educational workers; OEW: other essential workers.

Results are presented as weighted counts, weighted percentages with 95 % confidence intervals, or weighted means with standard deviations.

### 3.2 Compositional differences between groups

Substantial compositional differences were observed across gender and migration background groups (Table 1) and confirmed by the model-based average predicted probabilities (Table 2).

**Table 2:** Average predicted probabilities of being a non-essential worker or low- or high-class essential worker by gender and migration background groups and overall.

Essential worker	Occupational class	Men without a migration background	Women without a migration background	Men with a migration background	Women with a migration background	All
Non-essential worker		77.6^a^ [76.8–78.3]	57.5 [56.6–58.4]	78.2 [76.7–79.7]	60.6 [58.7–62.5]	66.5 [65.9–67]
HCW	High-class	2.4 [2.1–2.6]	8.1 [7.6–8.6]	1.7 [1.2–2.1]	5.2 [4.3–6.0]	5.4 [5.1–5.6]
	Low-class	1.3 [1.1–1.5]	7.4 [6.9–7.8]	1.4 [1.0–1.8]	8.7 [7.6–9.9]	4.9 [4.6–5.1]
SEW	High-class	4.2 [3.8–4.5]	9.6 [9.0–10.1]	3.0 [2.4–3.6]	5.3 [4.4–6.2]	6.8 [6.5–7.1]
	Low-class	0.6 [0.5–0.8]	9.3 [8.8–9.9]	0.8 [0.5–1.2]	11.5 [10.2–12.8]	5.7 [5.5–6]
OEW	High-class	3.2 [2.9–3.5]	1.3 [1.1–1.5]	1.5 [1.1–1.9]	1.0 [0.7–1.4]	2.0 [1.9–2.2]
	Low-class	10.7 [10.2–11.3]	6.8 [6.3–7.2]	13.3 [12.1–14.6]	7.7 [6.6–8.7]	8.7 [8.4–9]
Total	100	100	100	100	100	

HCW: healthcare workers; SEW: social and educational workers; OEW: other essential workers.

Average predicted probabilities were estimated from a weighted multinomial logistic regression.

The model is adjusted for age, living in a densely populated neighborhood, and living in overcrowded housing.

The explanatory variable categories are displayed in the columns, and the outcome variable categories in the rows to maintain consistency with other tables and make the results easier to follow.

a Example interpretation: 77.6 % of men without a migration background are predicted to be non-essential workers.

Compared to men without a migration background, women without a migration background were more likely to be HCWs and SEWs, particularly in low-class positions. Women with a migration background showed a similar pattern, although their representation in high-class SEWs did not differ significantly. Both groups of women also had lower probabilities of belonging to either of the OEW categories compared to the men without a migration background. Among men, those with a migration background were significantly less likely than those without a migration background to occupy any high-class essential job, and more likely to be low-class OEWs (Table 2).

When comparing women without a migration background with men with a migration background, the latter had markedly lower representation among HCWs and SEWs and a higher probability of being low-class OEWs. Among women, those without a migration background had higher representation in high-class HCW and high-class SEW jobs, whereas women with a migration background were more likely to occupy low-class SEW positions (Table 2).

### 3.3 Association differences between groups

In the overall sample, both high- and low-class HCW statuses were significantly associated with increased SARS-CoV-2 seropositivity compared to non-essential workers. In the stratified analysis, this pattern persisted among women without a migration background. Among women with a migration background, elevated seropositivity was observed not only for both HCW categories but also for those in low-class SEW and low-class OEW jobs. In contrast, no significant associations were found between the different classes of essential worker jobs and infection risk in either male stratum (Table 3).

**Table 3:** Association between being a high-class or low-class essential worker and SARS-CoV-2 infection in each gender and migration background stratum and overall.

Essential worker	Occupational class	Men without a migration background		Women without a migration background		Men with a migration background		Women with a migration background		All	
		% positive [CI]	OR [CI]	% positive [CI]	OR [CI]	% positive [CI]	OR [CI]	% positive [CI]	OR [CI]	% positive [CI]	OR [CI]
Non-essential worker	8.7 [8.1–9.3]	Reference	9.4 [8.8–10.1]	Reference	13.2 [11.5–15]	Reference	11.8 [10.2–13.5]	Reference	9.8 [9.4–10.2]	Reference	
HCW	High-class	12.4 [8.7–16.9]	1.4 [0.89–2.22]	15.9 [13.9–18.1]	1.82^a^ [1.49–2.22]	18.1 [6.9–35.4]	1.46 [0.64–3.36]	20.5 [13.9–28.5]	1.87 [1.06–3.3]	15.7 [13.9–17.7]	1.72 [1.44–2.04]
	Low-class	6.4 [3–11.6]	0.77 [0.32–1.82]	15.0 [13–17.3]	1.73 [1.32–2.27]	18.4 [6.1–38.4]	1.34 [0.35–5.14]	26.4 [20.3–33.3]	2.70 [1.47–4.99]	16 [14–18.1]	1.76 [1.38–2.23]
SEW	High-class	9.1 [6.6–12.1]	1.07 [0.68–1.68]	10.6 [9–12.3]	1.13 [0.92–1.38]	13.1 [5.5–25.1]	1.05 [0.51–2.19]	13.6 [8.1–20.9]	1.18 [0.71–1.97]	10.5 [9.2–12]	1.11 [0.93–1.33]
	Low-class	6.6 [2–15.5]	0.73 [0.26–2.06]	9.3 [7.8–11]	1.02 [0.78–1.35]	9.3 [0.5–36.1]	0.55 [0.05–6.28]	22.8 [17.7–28.6]	2.42 [1.38–4.22]	11.7 [10.1–13.5]	1.24 [0.96–1.58]
OEW	High-class	10.7 [7.7–14.3]	1.28 [0.83–1.97]	9.6 [5.9–14.6]	0.98 [0.46–2.1]	21.3 [8.7–39.7]	1.87 [0.51–6.84]	13.0 [3–32.2]	1.27 [0.35–4.55]	11.4 [9.1–14.1]	1.27 [0.88–1.84]
	Low-class	8.2 [6.7–9.9]	1.02 [0.73–1.41]	8.1 [6.5–10]	0.88 [0.6–1.29]	14 [9.8–19.2]	1.13 [0.53–2.44]	25.8 [19.2–33.4]	2.83 [1.08–7.38]	10.2 [9.1–11.4]	1.12 [0.86–1.46]

HCW: healthcare workers; SEW: social and educational workers; OEW: other essential workers.

Bolded results are statistically significant at the 5 % level.

Results are presented as weighted seropositivity percentages with 95 % confidence intervals, along with relative risks derived from five weighted logistic regression models (one for each stratum and one for the overall study sample).

The models are adjusted for age, living in a densely populated neighborhood and living in overcrowded housing.

a Example interpretation: Among women without a migration background, high-class healthcare workers have 1.82 times higher odds of having positive SARS-CoV-2 serology compared to non-essential workers.

## 4 Decomposition of gender and migration background inequalities

Tables 4 and 5 present decomposition analyses of SARS-CoV-2 seropositivity gaps by gender and migration background. In Table 4, the first row summarizes the seropositivity differences between men without a migration background and the three other gender and migration background groups, while the first row of Table 5 focuses on the gap between women without a migration background and men and women with a migration background. In both tables, these gaps are then decomposed into contributions from compositional differences (unequal representation in essential worker groups) and association differences (variation in infection risk within similar essential worker groups).

**Table 4:** Decomposition of the SARS-CoV-2 seropositivity difference between men without a migration background and other gender and migration background groups into contributions from compositional differences and association differences.

		Women without a migration background		Men with a migration background		Women with a migration background	
Percentage point difference in SARS-CoV-2 seropositivity relative to men without a migration background		1.6		4.7		6.9	
		Compositional contribution	Association-intensity contribution	Compositional contribution	Association-intensity contribution	Compositional contribution	Association-intensity contribution
Due to essential worker status and occupational class							
HCW	High-class	+22.6^a^	+3.6	−0.6	+0.3	+4.0	+1.4
	Low-class	+21.1	+5.8^b^	+0.0	+2.0	+12.8	+2.6
SEW	High-class	+4.7	+2.2	−0.1	−0.2	+0.5	+1.1
	Low-class	+0.3	+0.9	−0.2	−0.4	+14.7	+1.0
OEW	High-class	+0.2	−4.7	−2.5	+3.8	−1.0	−0.0
	Low-class	+3.6	−8.3	+0.4	+3.8	−7.0	+15.8
Due to covariates	−0.7	−5.4	+62.7	+63.8	+24.8	+6.6	
Residual unexplained components		+54.1		−32.8		+22.6	

HCW: healthcare workers; SEW: social and educational workers; OEW: other essential workers.

Bolded results are statistically significant at the 5 % level.

All estimates are weighted.

Covariates: Living in a densely populated neighborhood and living in overcrowded housing.

Compositional contribution: the share of the seropositivity gap explained by unequal distributions of essential-worker statuses across groups (compositional differences).

Association-intensity contribution: the share of the seropositivity gap due to group-specific differences in the intensity of the association between essential-worker status and seropositivity (association differenceswmen and men with a migration background).

a Example interpretation: The overrepresentation of women without a migration background among the high-class HCWs explains 22.6 % of the seropositivity difference between women without a migration background and men without a migration background.

b Example interpretation: Stronger association between low-class HCW status and SARS-CoV-2 seropositivity in women without a migration background compared to men without a migration background explains 5.8 % of the seropositivity difference between these two groups.

**Table 5:** Decomposition of the SARS-CoV-2 seropositivity difference between women without a migration background and men and women with a migration background into contributions from compositional differences and association differences.

		Men with a migration background		Women with a migration background	
Percentage point difference in SARS-CoV-2 seropositivity relative to women without a migration background		3.1		5.3	
		Compositional contribution	Association-intensity contribution	Compositional contribution	Association-intensity contribution
Due to essential worker status and occupational class					
HCW	High-class	−8.0	+2.5	−5.3^a^	+2.1
	Low-class	−4.9	+2.8	+1.7	+7.9
SEW	High-class	−0.5	+1.4	−2.3	+1.6
	Low-class	+14.1	+6.3	+2.8	+16.3^b^
OEW	High-class	+0.5	−1.2	−0.2	+0.8
	Low-class	+2.1	−2.4	+0.1	+17.1
Due to covariates	+90.1	+30.9	+34.1	+12.5	
Residual unexplained components		+28.0		+10.8	

HCW: healthcare workers; SEW: social and educational workers; OEW: other essential workers.

Bolded results are statistically significant at the 5 % level.

All estimates are weighted.

Covariates: Living in a densely populated neighborhood and living in overcrowded housing.

Compositional contribution: the share of the seropositivity gap explained by unequal distributions of essential-worker statuses across groups (compositional differences).

Association-intensity contribution: the share of the seropositivity gap due to group-specific differences in the intensity of the association between essential-worker status and seropositivity (association differences).

a Example interpretation: The fact that the women with a migration background are underrepresented among high-class HCWs compared to the women without a migration background has reduced the seropositivity percentage point difference between these two groups by 5.3 .%

b Example interpretation: Stronger association between low-class SEW status and SARS-CoV-2 seropositivity in women with a migration background compared to women without a migration background explains 16.3 % of the seropositivity difference between these two groups.

Among individuals without a migration background, women had a 1.6-percentage-point higher SARS-CoV-2 seropositivity rate than men. Non-linear decomposition analysis revealed that this gap was influenced by differences in occupational composition as well as by differences in the association between essential worker status and infection risk. Specifically, equalizing women without a migration background's representation in high-class and low-class HCW jobs with men without a migration background would reduce the gap by 22.6 % and 21.1 %, respectively. Additionally, the stronger association between low-class HCW jobs and seropositivity among women without a migration background compared to men from the same migration-background group accounted for a further 5.8 % of the gap (Table 4).

Women with a migration background exhibited the highest SARS-CoV-2 seropositivity, with a 6.9 percentage-point difference relative to men without a migration background. This disparity was largely due to their occupational composition, including their overrepresentation in high-class HCW jobs (4.0 %), low-class HCW jobs (12.8 %), and low-class SEW jobs (14.7 %). In addition, stronger associations between low-class essential occupations and seropositivity among women with a migration background further contributed to the gap, accounting for an extra 2.6 % for low-class HCWs, 1.0 % for low-class SEWs, and 15.8 % for low-class OEWs (Table 4).

The SARS-CoV-2 seropositivity gap of 5.3 percentage points between women with and without a migration background was influenced by nearly offsetting compositional effects and substantial differences in associations. Compositional differences contributed positively to the gap due to women with a migration background's overrepresentation in low-class HCW (1.7 %), low-class SEW (2.8 %), and low-class OEW (0.1 %) jobs, but this was partially offset by their underrepresentation in high-class HCW roles, which reduced the gap by 5.3 %, resulting in a small net negative compositional effect. Additionally, stronger associations between infection and low-class SEW and OEW jobs among women with a migration background accounted for 16.3 % and 17.1 % of the gap, respectively (Table 5).

Men with a migration background had a higher SARS-CoV-2 seropositivity than both men and women without a migration background (by 4.7 and 3.1 percentage points, respectively). Neither gap was attributable to essential worker status; instead, both resulted primarily from differences in covariate distributions (densely populated neighborhoods, overcrowded housing) and their higher associations with infection risk (Tables 4 and 5).

### 4.1 Sensitivity analysis

Using a stricter seropositivity definition (ELISA ≥ 1.1), the infection rate gradient persisted: 5.6 % for men without a migration background, 6.3 % for women without a migration background, 10.1 % for men with a migration background, and 11.5 % for women with a migration background. However, the gender gap within the individuals without a migration background was no longer statistically significant (Supplementary Table 2). Decomposition analyses of significant gaps closely mirrored primary analysis results (Supplementary Tables 3–4).

Repeating the analysis with an alternative migration background classification yielded larger seroprevalence gaps: 8.8 % in invisible-minority men or men without a migration background, 10.5 % in invisible-minority women or women without a migration background, 16.7 % in FOD-native/visible-minority men, and 19.2 % in FOD-native/visible-minority women (Supplementary Table 5). Decomposition showed that the gender gap within the groups without a migration background or belonging to the invisible-minority category resulted from women's overrepresentation in HCW jobs and the stronger association between low-class HCW occupations and infection risk. The gap between men without a migration background or belonging to the invisible-minority category and FOD-native/visible-minority women was due to the latter group's concentration in low-class essential jobs and the stronger association of these jobs with seropositivity. Similarly, the gap between women without a migration background or belonging to the invisible-minority category and FOD-native/visible-minority women was explained by the latter group's overrepresentation in low-class essential jobs and increased infection risk in low-class non-HCW occupations (Supplementary Tables 6–7).

## 5 Discussion

### 5.1 Main results

By the end of 2020, SARS-CoV-2 seroprevalence among workers in France showed a clear gradient: lowest among men without a migration background, higher among women without a migration background, and highest among workers with a migration background.

Women experienced a dual burden exacerbated by their migration background: disproportionate representation in specific essential jobs and higher infection risk within those occupations. Both women with and without a migration background were overrepresented among healthcare workers, and women with a migration background were additionally concentrated in low-class social and educational jobs, driving their increased seropositivity compared to men without a migration background. Their higher seroprevalence also stemmed from stronger infection associations with low-class healthcare jobs among women without a migration background, and across all low-class essential occupations for women with a migration background. Comparing women with and without a migration background revealed additional intersectional inequalities: although compositional differences nearly offset each other, women with a migration background faced notably higher infection risk within low-class social and educational jobs and low-class other essential occupations, further increasing their seropositivity

### 5.2 Interpretation

Our study extends earlier work on gender and migration background disparities in SARS-CoV-2 infection (Doerre and Doblhammer, 2022; Jaljaa et al., 2022) by showing how these factors intersect to shape infection risk in complex ways. Although rarely explored for infection (Rani et al., 2025), this intersection has been studied for mortality. During the first wave of the pandemic in France, excess mortality was higher among non-European-born men and women, particularly those aged 40–69 years from sub-Saharan Africa, North Africa, Asia, and Oceania, than among their native-born counterparts, although gender differences were not statistically significant (Khlat et al., 2022). In the United States, among adults aged 25 to 64, ethno-racial minority men with lower socioeconomic position had the highest COVID-19-related death rate in 2020 (Pathak et al., 2022). A comparable pattern was observed in England and Wales, where age-adjusted hazard ratios for COVID-19 death in spring 2020 were highest among Black and Bangladeshi/Pakistani men (Ayoubkhani et al., 2020).

Women are known to be overrepresented in the healthcare, social, and educational sectors (Hegewisch et al., 2014), a pattern previously linked to higher COVID-19 risk among working-age women (Doerre and Doblhammer, 2022). Our study adds nuance by differentiating between women with and without a migration background, as well as between high- and low-class essential occupations, and by quantifying how these distributions contribute to disparities in infection risk.

The gender and migration background profile of “other essential workers” (OEW) hinges on how the category is defined. In this study, we applied a context-calibrated OEW definition based on Mutambudzi et al. (Mutambudzi et al., 2021). In the UK, this definition identifies an OEW group that is predominantly male and foreign-born. Applying the same classification to France, we uncover a more nuanced pattern: men with a migration background are overrepresented in lower-class OEW jobs, whereas men without a migration background are more likely to occupy higher-class OEW positions. This origin-based divide mirrors broader European evidence that immigrant workers are disproportionately concentrated in lower-skilled essential occupations, such as cleaning and transport (Fasani and Mazza, 2020).

Few studies have explored variations in the association between essential occupations and COVID-19 risk. A systematic review on gender differences in occupation-specific infectious diseases reported that most studies found no gender differences in SARS-CoV-2 infection risk among patient-facing healthcare workers and physicians; however, two fair-quality studies identified higher risks among men (Biswas et al., 2024). Conversely, a pooled estimate based on two studies suggested higher infection risk among female healthcare workers compared to their male counterparts (Dzinamarira et al., 2022).

In Norway, during 2020, a register-based study of adults aged 20–70 examined selected occupations that were commonly held by immigrants and had high rates of notified COVID-19. Within each occupational group, immigrants had substantially higher odds of COVID-19 than Norwegian-born individuals. However, within country-of-birth groups, immigrants working in the selected occupations generally had similar odds of COVID-19 as those not in these occupations, with a few exceptions (Kjøllesdal and Magnusson, 2021). These findings indicate that the association between occupation and infection risk differs by country of birth. Our study observed stronger associations between certain essential occupations and COVID-19 risk among women, particularly among those with a migration background, contributing significantly to their higher seroprevalence.

The contribution of these association differences, often referred to as the “unexplained component” in decomposition analysis, highlights disparities that persist even when groups share identical observable characteristics. While disparities arising from workforce composition may be considered justifiable, the remaining gaps could reflect unmeasured factors, including discrimination or other structural disadvantages not captured in the model (Rahimi and Hashemi Nazari, 2021). Further research should clarify the mechanisms underlying job-specific differences in infection risk. Possible explanations include differential on-site exposure factors (e.g., availability, fit, and effective use of personal protective equipment, or variations in tasks performed leading to different levels of face-to-face contact and exposure to infectious agents), off-site work-related exposures (e.g., type of commuting - crowded public transport versus private vehicle), and broader structural inequalities (e.g., legal insecurity, precarious employment contracts, and limited access to labor rights). These factors may vary systematically across gender and migration-background groups holding similar jobs, thereby contributing to disparities in infection risk.

In contrast to these occupation-linked disparities, the entire seropositivity gap between men with and without a migration background was driven by factors other than essential-work status, probably because men with a migration background constitute the smallest share of essential workers.

Our decomposition analysis identified substantial unexplained residual components, indicating essential-worker status as just one of the exposure mechanisms contributing to gender and migration background inequalities in COVID-19 risk. Beyond controlled covariates (overcrowded housing, densely populated neighborhoods), other non-occupational exposures, such as unpaid care responsibilities, may differ systematically by gender and migration background. Occupational exposure of other household members might also indirectly elevate some essential workers' infection risk. Indeed, a study from Ontario, Canada, found that attributing secondary household cases to occupational exposure increased workplace COVID-19 outbreak burdens by 56 % (Murti et al., 2021). Thus, our findings represent only a partial estimate of the total contribution of occupational factors to infection inequalities.

Migration-related inequalities may have shifted after the first year of the pandemic and the introduction of vaccines. In France, ethno-racial minorities were more reluctant to receive COVID-19 vaccination, partly due to institutional mistrust shaped by past experiences of structural discrimination(Bajos et al., 2022) Conversely, these groups also showed greater adherence to the public health measures (Gosselin et al., 2022), which may have helped mitigate their risk despite their higher exposure.

The role of essential work in infection risk has also likely changed. While the earlier phases of the pandemic were marked by elevated infection risks among workers in healthcare, transport, and food production, these risks declined over time. In contrast, persistently high infection rates were observed in the education and social care sectors (Rhodes et al., 2024). In Canada, community and household exposures became the primary sources of infection for healthcare workers during the Omicron wave, in contrast to earlier waves when occupational exposure was more prominent (Martinez-Cajas et al., 2025). These shifts underscore the impact of evolving institutional and community-level interventions on pandemic-related inequalities.

### 5.3 Strengths and limitations

A significant strength of this study was its joint analysis of gender, migration background, essential-worker status, and occupational class, thereby providing an explicitly intersectional perspective on SARS-CoV-2 infection risk. Moreover, the large, nationally representative sample allowed us to comprehensively investigate the working population. Additionally, consistent results across sensitivity analyses further confirmed the robustness of our findings. Finally, the infection was ascertained serologically rather than through self-report or routine testing, thus minimizing potential biases related to unequal healthcare access and health literacy.

Nevertheless, our work had limitations. First, we were unable to disaggregate within the broad HCW, SEW, and OEW categories, which likely mask important internal heterogeneity. Some of the observed association differences may therefore reflect residual compositional variation. For instance, nursing assistants (mostly women) and ambulance drivers (mostly men) are both categorized as lower-class HCWs. Therefore, the apparent gender difference in COVID-19 risk among lower-class HCWs may partly reflect these internal compositional variations. This limitation is even more pronounced in the second sensitivity analysis, which uses the alternative migration background variable, where small sample sizes necessitate merging SEW and OEW occupations - two groups that differ considerably in sociodemographic composition, including gender.

Second, the sample was not large enough to distinguish between immigrants and their descendants or to examine differences by geographic origin, despite evidence from other studies showing significant disparities in COVID-19-related mortality (Khlat et al., 2022) and occupational exposures (Ghoroubi et al., 2022) according to migration generation and geographic region of origin.

Third, relying on a binary sex variable as a proxy for gender prevented us from capturing the full spectrum of gender identities. As most COVID-19 research treats gender as binary (Biswas et al., 2024), the impact of assigning non-binary individuals to either the male or female category remains unclear and may introduce bias.

In addition, antibody persistence is limited and may decline five to seven months after infection (Ripperger et al., 2020), suggesting that our study may underestimate early 2020 infections, when no mitigation measures were yet in place.

Finally, our findings may underestimate the vulnerability of immigrant workers because, like other register-based studies, EpiCoV could not include asylum seekers and undocumented migrant workers, groups shown to experience higher COVID-19 exposure and infection rates (Mengesha et al., 2022).

## 6 Conclusion

Women faced a dual burden contributing to their elevated SARS-CoV-2 infection risk at the beginning of the pandemic, further exacerbated by their migration background. They were disproportionately represented in certain essential occupations and experienced increased infection risk when occupying specific lower-class essential jobs. Awareness of these intersectional mechanisms is critical for developing targeted public health strategies that effectively protect individuals experiencing multiple layers of social disadvantage and occupational exposures.

## Ethics statement

EpiCoV survey was approved by an ethics committee (Comité de Protection des Personnes Sud Méditerranée III 2020-A01191–38) and France's National Data Protection Agency (Commission Nationale Informatique et Liberte's, CNIL, MLD/MFI/AR205138) in April 2020.

## Funding

This work was supported by the French Institute for Demographic Studies (INED) and the Gender and Health Inequalities (GENDHI) project (ERC-2019-SyG), which has received funding from the European Research Council (ERC) under the European Union’s Horizon 2020 research and innovation programme (grant agreement No. 856,478), as part of a doctoral thesis. The funders were not involved in the study design, the collection, analysis, and interpretation of the data, the writing of the report, and the decision to submit the paper for publication.

## Data availability statement

The EpiCoV study data are available for research purposes upon approval by the French Ethics and Regulatory Committees (Comité du Secret Statistique, CESREES, and CNIL). Information on the access procedure can be found on the CASD website (https://www.casd.eu/).

## Declaration of generative AI and AI-assisted technologies in the writing process

During the preparation of this work the authors used ChatGPT (OpenAI), DeepL, and Grammarly in order to assist with language editing, rephrasing, and grammar checks. After using these tools, the authors reviewed and edited the content as needed and take full responsibility for the content of the published article.

## Financial disclosure statement

This work was supported by the French Institute for Demographic Studies (INED) and the Gender and Health Inequalities (GENDHI) project (ERC-2019-SyG), which has received funding from the European Research Council (ERC) under the European Union’s Horizon 2020 research and innovation programme (grant agreement No. 856,478), as part of a doctoral thesis. The funders were not involved in the study design, the collection, analysis, and interpretation of the data, the writing of the report, and the decision to submit the paper for publication.

## CRediT authorship contribution statement

**Narges GHOROUBI:** Writing – review & editing, Writing – original draft, Visualization, Validation, Software, Project administration, Methodology, Investigation, Formal analysis, Conceptualization. **Myriam KHLAT:** Writing – review & editing, Validation, Supervision, Project administration, Methodology, Investigation, Funding acquisition, Conceptualization. **Emilie COUNIL:** Writing – review & editing, Validation, Supervision, Resources, Project administration, Methodology, Investigation, Funding acquisition, Data curation, Conceptualization.

## Declaration of competing interest

The authors declare no conflicts of interest.

## Appendix Supplementary materials

Image, application 1
