# Supplementary material for: Essential worker status, gender, and migration background disparities in COVID-19: An intersectional approach
Source: J Migr Health. 2025 Dec 27;13:100392. doi: 10.1016/j.jmh.2025.100392 (PMC13404061; doi:10.1016/j.jmh.2025.100392)
Supplement: Supplementary file 1 [file mmc1.docx]

**Supplementary material**

*Supplementary Figure 1. Study sample selection from the EpiCoV cohort*

*Supplementary Table 1 Essential worker definition*

| PCS 2020 code and titles classified as healthcare workers | | | | PCS 2020 code and titles classified as social and educational workers | | | | PCS 2020 code and titles classified as other essential workers | | | |
| --- | --- | --- | --- | --- | --- | --- | --- | --- | --- | --- | --- |
| High class | | Low class | | High class | | Low class | | High class | | Low class | |
| 31A1 | Médecins libéraux spécialistes | 43C3 | Préparateurs en pharmacie | 34A1 | Enseignants titulaires du secondaire général et technologique | 42B2 | Surveillants scolaires et assistants d’éducation | 10A7 | Autres exploitants agricoles | 21E1 | Conducteurs de taxis, d'ambulances et autres entrepreneurs du transport individuel |
| 31A2 | Médecins libéraux généralistes | 52B3 | Agents de service des établissements de santé | 34A2 | Chefs d'établissement de l'enseignement secondaire, inspecteurs | 43D7 | Animateurs socio-culturels et de loisirs | 23B2 | Chefs d’entreprise de l’agriculture, de l’industrie et des transports, de 11 à 49 personnes | 21E2 | Transporteurs indépendants (routiers, fluviaux, maritimes et aériens) |
| 31A3 | Chirurgiens-dentistes | 52C1 | Aides-soignants | 34B1 | Enseignants du supérieur | 52C3 | Auxiliaires de puériculture | 33D1 | Officiers des Armées et de la Gendarmerie (sauf grades de colonels, généraux et équivalents) | 21E5 | Artisans du nettoyage, de la récupération et des services divers |
| 31A6 | Pharmaciens libéraux | 52C2 | Assistants de professions médicales et paramédicales | 42A1 | Enseignants titulaires du primaire | 52C4 | Agents spécialisés de crèche et des écoles maternelles | 37D1 | Directeurs de magasins et cadres de l’exploitation du commerce de détail | 22A1 | Commerçants de produits alimentaires |
| 34C1 | Médecins salariés hospitaliers | 52C5 | Aides médico-psychologiques et professions assimilées | 42A2 | Enseignants titulaires du secondaire professionnel | 56C1 | Assistants familiaux, familles d'accueil | 45B1 | Personnels de commandement de la Police nationale et de l’administration pénitentiaire | 52A3 | Employés administratifs de la fonction publique à technicité spécifique |
| 34C3 | Internes en médecine, odontologie et pharmacie | 52C6 | Ambulanciers | 42A3 | Enseignants non titulaires du primaire et du secondaire | 56C2 | Assistants maternels agréés | 46B2 | Responsables (non cadres) d'un secteur ou d'un rayon dans un commerce de détail | 52A4 | Facteurs et employés du service universel du courrier |
| 34C4 | Pharmaciens salariés |  |  | 42B1 | Conseillers principaux d'éducation | 56C3 | Autres salariés de particuliers pour la garde d’enfants | 47A1 | Techniciens de l’agriculture, de l’aquaculture, des forêts et de la protection de l’environnement | 52B1 | Agents de service de nettoyage (État, collectivités territoriales) |
| 43A1 | Cadres de santé |  |  | 43D1 | Directeurs et cadres du travail social et de l'animation socio-culturelle | 56D1 | Auxiliaires de vie sociale | 53A1 | Personnels d’encadrement et d’application de la Police nationale | 53A3 | Personnels d’encadrement et d’application de l’administration pénitentiaire |
| 43A2 | Sages-femmes |  |  | 43D2 | Assistants de service social, conseillers en économie sociale et familiale | 56D2 | Autres employés d’aide à domicile et accueillants familiaux | 53B1 | Gendarmes | 55A1 | Vendeurs en produits alimentaires |
| 43A3 | Infirmiers libéraux |  |  | 43D3 | Éducateurs spécialisés |  |  | 53C1 | Pompiers | 55B4 | Autres vendeurs spécialisés (carburant, tabac, presse) |
| 43A4 | Infirmiers spécialisés salariés |  |  | 43D4 | Moniteurs éducateurs |  |  | 54C1 | Contrôleurs et agents d'accompagnement des transports et du tourisme | 55D1 | Caissiers du commerce |
| 43A5 | Infirmiers en soins généraux salariés |  |  | 43D5 | Éducateurs techniques spécialisés, moniteurs d’ateliers |  |  | 65D1 | Conducteurs de trains, matelots de la marine marchande | 55D2 | Employés de libre-service du commerce |
| 43B1 | Masseurs-kinésithérapeutes rééducateurs |  |  | 43D6 | Éducateurs de jeunes enfants |  |  |  |  | 62C1 | Ouvriers qualifiés de l’agroalimentaire |
| 43B2 | Spécialistes de la rééducation de la motricité, du langage et de la vue |  |  |  |  |  |  |  |  | 62C4 | Ouvriers qualifiés de l’eau, de l’énergie et du traitement des déchets |
| 43C1 | Techniciens médicaux |  |  |  |  |  |  |  |  | 63A1 | Ouvriers des parcs et jardins |
|  |  |  |  |  |  |  |  |  |  | 64A1 | Conducteurs routiers de transport en commun |
|  |  |  |  |  |  |  |  |  |  | 64B1 | Conducteurs de poids lourds |
|  |  |  |  |  |  |  |  |  |  | 64B3 | Coursiers, livreurs, distributeurs à domicile |
|  |  |  |  |  |  |  |  |  |  | 67E3 | Agents peu qualifiés des services d'exploitation des transports |
|  |  |  |  |  |  |  |  |  |  | 68D1 | Ouvriers du nettoyage |
|  |  |  |  |  |  |  |  |  |  | 68D2 | Ouvriers peu qualifiés de l’assainissement et du traitement des déchets |
|  |  |  |  |  |  |  |  |  |  | 69A1 | Ouvriers agricoles de grandes cultures et de polyculture-élevage |
|  |  |  |  |  |  |  |  |  |  | 69A3 | Ouvriers de la viticulture et de l’arboriculture fruitière |
|  |  |  |  |  |  |  |  |  |  | 69A4 | Ouvriers de l’élevage |

PCS: Professions et catégories socioprofessionnelles (Occupations and socio-occupational categories in France)

*Supplementary Table 2 SARS-CoV-2 seropositivity rates in gender and migration background groups using stricter case definition (ELISA>=1.1)*

| Variable | | Men without a migration background  N= 11049 | Women without a migration background  N= 14715 | men without a migration backgroundMen with a migration background  N= 1910 | women without a migration backgroundWomen with a migration background  N= 2344 | All  N= 30018 | |
| --- | --- | --- | --- | --- | --- | --- | --- |
|  |  | % [CI] /  Mean (SD) | % [CI] /  Mean (SD) | % [CI] /  Mean (SD) | % [CI] /  Mean (SD) | N | %[CI] /  Mean (SD) |
| Seropositivity | No | 94.4  [93.9-94.8] | 93.7  [93.3-94.1] | 89.9  [88.4-91.2] | 88.5  [87.1-89.7] | 27938 | 93.1  [92.8-93.4] |
|  | Yes | 5.6  [5.2-6.1] | 6.3  [5.9-6.7] | 10.1  [8.8-11.6] | 11.5  [10.3-12.9] | 2080 | 6.9  [6.6-7.2] |

Results are presented as weighted counts or weighted percentages with 95% confidence intervals.

*Supplementary Table 3 Decomposition of the SARS-CoV-2 seropositivity difference between men without a migration background and men and women with a migration background into contributions from compositional differences and association differences using stricter case definition (ELISA>=1.1)*

|  | | men without a migration backgroundMen with a migration background | | women without a migration backgroundWomen with a migration background | |
| --- | --- | --- | --- | --- | --- |
| Percentage point difference in SARS-CoV-2 seropositivity relative to men without a migration background | | 4.5 | | 5.9 | |
|  | | Compositional contribution | Association-intensity contribution | Compositional contribution | Association-intensity contribution |
| Due to essential worker status and occupational class | | | | | |
| HCW | High-class | +0.2 | -4.3 | **+3.8^a^** | +0.7 |
|  | Low-class | -0.0 | +0.2 | **+11.9** | **+2.4^b^** |
| SEW | High-class | -0.1 | +0.3 | +0.6 | +1.7 |
|  | Low-class | -0.4 | -0.8 | **+16.0** | **+1.4** |
| OEW | High-class | +1.1 | -5.9 | -0.6 | -1.1 |
|  | Low-class | +0.5 | +2.6 | -7.7 | **+17.0** |
| Due to covariates | | +55.7 | +68.8 | +27.3 | +14.7 |
| Residual unexplained components | |  | -18.2 |  | +11.7 |

HCW: healthcare workers; SEW: social and educational workers; OEW: other essential workers.

Bolded results are statistically significant at the 5% level.

All estimates are weighted.

Covariates: Living in a densely populated neighborhood and living in overcrowded housing.

Compositional contribution: the share of the seropositivity gap explained by unequal distributions of essential-worker statuses across groups (compositional differences)

Association-intensity contribution: the share of the seropositivity gap due to group-specific differences in the intensity of the association between essential-worker status and seropositivity (association differences)

^a^ Example interpretation: The overrepresentation of women without a migration backgroundwomen with a migration background among the high-class HCWs explains 3.8% of the seropositivity difference between women without a migration backgroundwomen with a migration background and men without a migration background.

^b^ Example interpretation: Stronger association between low-class HCW status and SARS-CoV-2 seropositivity in women without a migration backgroundwomen with a migration background compared to men without a migration background explains 2.4% of the seropositivity difference between these two groups.

*Supplementary Table 4 Decomposition of the SARS-CoV-2 seropositivity difference between women without a migration background and men and women with a migration background into contributions from compositional differences and association differences using stricter case definition (ELISA>=1.1)*

|  | | men without a migration backgroundMen with a migration background | | women without a migration backgroundWomen with a migration background | |
| --- | --- | --- | --- | --- | --- |
| Percentage point difference in SARS-CoV-2 seropositivity relative to women without a migration background | | 3.8 | | 5.2 | |
|  | | Compositional contribution | Association-intensity contribution | Compositional contribution | Association-intensity contribution |
| Due to essential worker status and occupational class | |  | | | |
| HCW | High-class | +2.1 | +45.2 | **-4.5^a^** | +1.8 |
|  | Low-class | +1.6 | +44.7 | **+1.4** | +3.8 |
| SEW | High-class | -0.3 | +8.4 | -2.5 | +2.1 |
|  | Low-class | +19.5 | +81.8 | **+2.8** | **+14.0^b^** |
| OEW | High-class | -0.2 | +6.5 | -0.1 | -0.4 |
|  | Low-class | +1.7 | -26.9 | **+0.1** | **+18.3** |
| Due to covariates | | +56.0 | -186.8 | +33.5 | +14.6 |
| Residual unexplained components | |  | +46.8 |  | +15.1 |

HCW: healthcare workers; SEW: social and educational workers; OEW: other essential workers.

Bolded results are statistically significant at the 5% level.

All estimates are weighted.

Covariates: Living in a densely populated neighborhood and living in overcrowded housing.

Compositional contribution: the share of the seropositivity gap explained by unequal distributions of essential-worker statuses across groups (compositional differences)

Association-intensity contribution: the share of the seropositivity gap due to group-specific differences in the intensity of the association between essential-worker status and seropositivity (association differences)

^a^ Example interpretation: The fact that the women without a migration backgroundwomen with a migration background are underrepresented among high-class HCWs compared to the women without a migration background has reduced the seropositivity percentage point difference between these two groups by 4.5 %

^b^ Example interpretation: Stronger association between low-class SEW status and SARS-CoV-2 seropositivity in women without a migration backgroundwomen with a migration background compared to women without a migration background explains 14.0% of the seropositivity difference between these two groups.

*Supplementary Table 5 Baseline characteristics of the study population in gender and migration background distinguishing ethno-racial minorities from others*

| Variable | | | Men without a migration background or belonging to the invisible-minority group  N= 12069 | Women without a migration background or belonging to the invisible-minority group  N= 16028 | FOD natives or visible minority men  N= 890 | FOD natives or visible minority women  N= 1031 | All  N= 30018 | |
| --- | --- | --- | --- | --- | --- | --- | --- | --- |
|  |  |  | % [CI] /  Mean (SD) | % [CI] /  Mean (SD) | % [CI] /  Mean (SD) | % [CI] /  Mean (SD) | N | %[CI] /  Mean (SD) |
| Share of participants | | | 45.0  [44.2-45.9] | 43.9  [43.1-44.7] | 6.4  [5.7-7.1] | 4.7  [4.3-5.1] |  | 100 |
| Essential worker and class | Non-essential worker | | 78.3  [77.5-79] | 58.1  [57.4-58.9] | 76.4  [73.5-79.2] | 62.0  [59-65] | 20576 | 68.5  [68-69.1] |
|  | HCW | High-class | 2.4  [2.1-2.7] | 8.2  [7.8-8.6] | 1.9  [1.1-3] | 5.0  [3.7-6.5] | 1507 | 5.0  [4.8-5.3] |
|  |  | Low-class | 1.3  [1.1-1.5] | 7.3  [6.9-7.7] | 1.8  [1-2.8] | 10.0  [8.2-12] | 1309 | 4.4  [4.1-4.6] |
|  | Non-HCW | High-class | 7.1  [6.7-7.6] | 10.4  [10-10.9] | 4.2  [3-5.8] | 5.5  [4.2-7] | 2498 | 8.3[8-8.6] |
|  |  | Low-class | 10.9  [10.4-11.5] | 15.9  [15.4-16.5] | 15.7  [13.3-18.2] | 17.6  [15.3-20.1] | 4128 | 13.8[13.4-14.1] |
| Living in a densely populated neighborhood | No | | 65.4  [64.5-66.2] | 65.5  [64.7-66.2] | 33.1  [30-36.3] | 28.6  [25.9-31.5] | 18504 | 61.6  [61.1-62.2] |
|  | Yes | | 34.6  [33.8-35.5] | 34.5  [33.8-35.3] | 66.9  [63.7-70] | 71.4  [68.5-74.1] | 11514 | 38.4  [37.8-38.9] |
| Living in an overcrowded housing | No | | 92.6  [92.1-93] | 93.5  [93.1-93.9] | 70.2  [67.1-73.2] | 78.4  [75.8-80.9] | 27284 | 90.9  [90.6-91.2] |
|  | Yes | | 7.4  [7-7.9] | 6.5  [6.1-6.9] | 29.8  [26.8-32.9] | 21.6  [19.1-24.2] | 2734 | 9.1  [8.8-9.4] |
| Age | | | 42.2 (11.3) | 42.7 (11.1) | 40.2 (11.4) | 41.1 (11.1) |  |  |
| Seropositivity | No | | 91.2  [90.7-91.7] | 89.5  [89-90] | 83.3  [80.7-85.7] | 80.8  [78.3-83.2] | 26852 | 89.5  [89.1-89.8] |
|  | Yes | | 8.8  [8.3-9.3] | 10.5  [10.0-11.0] | 16.7  [14.3-19.3] | 19.2  [16.8-21.7] | 3166 | 10.5  [10.2-10.9] |

HCW: healthcare workers; Non-HCW: non-healthcare workers.

Low-class social, educational, and other essential workers were grouped into a single low-class non-healthcare essential worker category; high-class workers from these sectors were similarly merged into a high-class non-healthcare essential worker category.

Results are presented as weighted counts, weighted percentages with 95% confidence intervals, or weighted means with standard deviations.

*Supplementary Table 6 Decomposition of the SARS-CoV-2 seropositivity difference between men without a migration background or belonging to the invisible-minority group, and other gender and migration background groups into contributions from compositional differences and association differences*

|  | | Women without a migration background or belonging to the invisible-minority group | | FOD natives or visible minority men | | FOD natives or visible minority women | |
| --- | --- | --- | --- | --- | --- | --- | --- |
| Percentage point difference in SARS-CoV-2 seropositivity relative to men without a migration background or belonging to the invisible-minority group | | 1.7 | | 7.9 | | 10.4 | |
|  | | Compositional contribution | Association-intensity contribution | Compositional contribution | Association-intensity contribution | Compositional contribution | Association-intensity contribution |
| Due to essential worker status and occupational class | |  | | | | | |
| HCW | High-class | **+22.3^a^** | +3.3 | -0.2 | -0.3 | +2.5 | +0.6 |
|  | Low-class | **+22.2** | **+6.1^b^** | +0.2 | +1.3 | **+12.9** | **+1.7** |
| Non-HCW | High-class | +2.9 | +1.2 | -2.0 | +3.7 | -0.4 | +0.3 |
|  | Low-class | +1.6 | +1.7 | -0.7 | -2.0 | **+10.0** | **+10.8** |
| Due to covariates | | -2.7 | -3.5 | +54.1 | +35.4 | +6.4 | -15.8 |
| Residual unexplained components | |  | +45.1 |  | +10.5 |  | **+66.7** |

HCW: healthcare workers; Non-HCW: non-healthcare workers.

Low-class social, educational, and other essential workers were grouped into a single low-class non-healthcare essential worker category; high-class workers from these sectors were similarly merged into a high-class non-healthcare essential worker category.

Bolded results are statistically significant at the 5% level.

All estimates are weighted.

Covariates: Living in a densely populated neighborhood and living in overcrowded housing.

Compositional contribution: the share of the seropositivity gap explained by unequal distributions of essential-worker statuses across groups (compositional differences)

Association-intensity contribution: the share of the seropositivity gap due to group-specific differences in the intensity of the association between essential-worker status and seropositivity (association differences)

^a^ Example interpretation: The overrepresentation of women without a migration background or belonging to the invisible-minority group among the high-class HCWs explains 22.3% of the seropositivity difference between them and the men with the same migration background .

^b^ Example interpretation: Stronger association between low-class HCW status and SARS-CoV-2 seropositivity in women without a migration background or belonging to the invisible-minority group compared to men with the same migration background explains 6.1% of the seropositivity difference between these two groups.

*Supplementary Table 7 Decomposition of the SARS-CoV-2 seropositivity difference between women without a migration background or belonging to the invisible-minority group and two FOD natives or visible minority groups into contributions from compositional differences and association differences*

|  | | FOD natives or visible minority men | | FOD natives or visible minority women | |
| --- | --- | --- | --- | --- | --- |
| Percentage point difference in SARS-CoV-2 seropositivity relative to women without a migration background or belonging to the invisible-minority group | | 6.2 | | 8.7 | |
|  | | Compositional contribution | Association-intensity contribution | Compositional contribution | Association-intensity contribution |
| Due to essential worker status and occupational class | |  | | | |
| HCW | High-class | -3.2 | -6.9 | -4.0 | -0.2 |
|  | Low-class | -3.4 | -4.7 | **+5.2^a^** | +3.6 |
| Non-HCW | High-class | -5.4 | +7.4 | -1.5 | +0.0 |
|  | Low-class | +0.0 | -5.4 | **+3.3** | **+20.9^b^** |
| Due to covariates | | +69.6 | +56.2 | +9.1 | -14.7 |
| Residual unexplained components | |  | -4.4 |  | **+78.3** |

HCW: healthcare workers; Non-HCW: non-healthcare workers.

Bolded results are statistically significant at the 5% level.

All estimates are weighted.

Covariates: Living in a densely populated neighborhood and living in overcrowded housing.

Compositional contribution: the share of the seropositivity gap explained by unequal distributions of essential-worker statuses across groups (compositional differences)

Association-intensity contribution: the share of the seropositivity gap due to group-specific differences in the intensity of the association between essential-worker status and seropositivity (association differences)

^a^ Example interpretation: The overrepresentation of FOD natives or visible minority women among the low-class HCWs explains 5.2% of the seropositivity difference between them and women without a migration background or belonging to the invisible-minority group.

^b^ Example interpretation: Stronger association between low-class non-HCW status and SARS-CoV-2 seropositivity in FOD natives or visible minority women compared to women without a migration background or belonging to the invisible-minority group explains 20.9% of the seropositivity difference between these two groups.
